# Hepatocyte growth factor is crucial for development of the carapace in turtles

**DOI:** 10.1111/j.1525-142X.2011.00474.x

**Published:** 2011-05

**Authors:** Yoshie Kawashima-Ohya, Yuichi Narita, Hiroshi Nagashima, Ryo Usuda, Shigeru Kuratani

**Affiliations:** Laboratory for Evolutionary Morphology, RIKEN Center for Developmental Biology (CDB)2-2-3 Minatojima-minami, Kobe 650-0047, Japan

## Abstract

Turtles are characterized by their shell, composed of a dorsal carapace and a ventral plastron. The carapace first appears as the turtle-specific carapacial ridge (CR) on the lateral aspect of the embryonic flank. Accompanying the acquisition of the shell, unlike in other amniotes, hypaxial muscles in turtle embryos appear as thin threads of fibrous tissue. To understand carapacial evolution from the perspective of muscle development, we compared the development of the muscle plate, the anlage of hypaxial muscles, between the Chinese soft-shelled turtle, *Pelodiscus sinensis*, and chicken embryos. We found that the ventrolateral lip (VLL) of the thoracic dermomyotome of *P. sinensis* delaminates early and produces sparse muscle plate in the lateral body wall. Expression patterns of the regulatory genes for myotome differentiation, such as *Myf5, myogenin, Pax3*, and *Pax7* have been conserved among amniotes, including turtles. However, in *P. sinensis* embryos, the gene *hepatocyte growth factor* (*HGF*), encoding a regulatory factor for delamination of the dermomyotomal VLL, was uniquely expressed in sclerotome and the lateral body wall at the interlimb level. Implantation of COS-7 cells expressing a HGF antagonist into the turtle embryo inhibited CR formation. We conclude that the de novo expression of *HGF* in the turtle mesoderm would have played an innovative role resulting in the acquisition of the turtle-specific body plan.

## INTRODUCTION

The turtle shell offers a number of curious and important problems for evolutionary developmental biology. Of these, the carapace or the dorsal moiety of the shell is notable. This structure mainly consists of laterally expanded ribs, which in normal tetrapods grow ventrally into the lateral body wall (reviewed by Nagashima et al. 2007). The carapace first appears at the late pharyngula stage in turtle development as a longitudinal ridge, the carapacial ridge (CR), on the lateral aspect of the flank (reviewed by Burke 1989, 1991), along the distal tips of the rib primordia. The CR is comprised of thickened ectoderm and underlying condensed mesenchyme, forming the leading edge of the carapacial primordium expanding marginally. Because of the histological similarity between the CR and the apical ectodermal ridge of limb buds, the CR has often been presumed to induce dorsolateral and superficial migration of rib precursor cells (Burke 1989, 1991, 2009; Gilbert et al. 2001, 2008; Loredo et al. 2001; Vincent et al. 2003; Cebra-Thomas et al. 2005; reviewed by Kuratani et al. 2011). However, our studies have shown that the CR functions in the rib growth not in a dorsoventral, but in a rostrocaudal direction, to form a flabellate pattern of ribs, through marginal growth of the carapacial primordium (Nagashima et al. 2007). This rib growth leads to encapsulation of the scapula by folding the lateral body wall (Nagashima et al. 2009).

As shown by mutant mouse models, amniote rib development largely depends on myotome development (Braun et al. 1992, 1994; Hasty et al. 1993; Braun and Arnold 1995; Patapoutian et al. 1995; Zhang et al. 1995; Grass et al. 1996; Yoon et al. 1997; Tremblay et al. 1998; Dickman et al. 1999; Henderson et al. 1999; Grifone et al. 2005; reviewed by Olson et al. 1996). Ribs are derived from intermyotomally positioned parts of the lateral sclerotome, termed the syndetome (Kato and Aoyama 1998; Huang et al. 2000; Brent et al. 2003, 2005; Evans 2003; reviewed by Brent and Tabin 2002; Christ et al. 2004; Christ and Scaal 2008;), induced by myotome through fibroblast growth factor (Patapoutian et al. 1995; Vinagre et al. 2010; also see Grass et al. 1996; Huang et al. 2003;) and platelet-derived growth factor signals (Soriano 1997; Tallquist et al. 2000;). These signaling molecules are induced by Myf5 and MRF4 in the myotome (Fraidenraich et al. 1998, 2000; also see Vinagre et al. 2010).

Turtle ribs develop initially with an anatomical pattern which is similar to that in other amniotes (Emelianov 1936; Nagashima et al. 2009;). However, they are arrested in the axial part of the embryonic body and never penetrate into the lateral body wall during elongation (Burke 1989; Nagashima et al. 2007;). Thus the turtle ribs are morphologically shorter than those in other amniotes. Concomitantly, the hypaxial muscle anlage in the turtle is also unique among amniotes in that it develops as a thin fibrous mass (Nagashima et al. 2005), implying a developmental relationship between the turtle-specific morphologies of ribs and muscle plates. Previously, we found that the *Myf5* gene, one of the myogenic regulatory factors (MRFs), shows a unique deletion of 12 sequential nucleotides specifically in turtles (Ohya et al. 2006), corresponding to the transactivation domain (Winter et al. 1992). Furthermore, two kinds of splicing variants of *Myf5* are found in Chinese soft-shelled turtles, *Pelodiscus sinensis* and hard-shelled red-eared slider, *Trachemys scripta*, of which the short form appears to function as a dominant negative form against the long one (Ohya et al. 2006). These findings imply that the axial arrest of the turtle ribs might be associated with the change in function of *Myf5*, because *Myf5* is implicated in the rib development of amniotes through muscle differentiation. However, its expression pattern is not consistent with this scenario, because expression of *Myf5* as well as another MRF, *MyoD*, was not observed in the ventral muscle plate of *P. sinensis*.

In this study, we first compared muscle plate development between chicken and turtle embryos at histological and molecular levels. We found that most of the gene expression patterns, including *Myf5*, are similar between the two species, underlining the importance of functional changes in *Myf5* in turtle rib development. Furthermore, we found turtle-specific expression patterns of *hepatocyte growth factor* (*HGF*), known to be involved in the formation of a subset of skeletal muscles. Unexpectedly, inhibition of HGF function leads to the arrest of CR development. These findings suggest complicated developmental changes in turtle evolution, which are also consistent with the morphology of a recently discovered intermediate fossil species, *Odontochelys* (Li et al. 2008).

## MATERIALS AND METHODS

### Embryos

Fertilized eggs of *P. sinensis* and chicken (*Gallus gallus*) were purchased from several local farms in Japan. The eggs were incubated at 30°C for turtle and 38°C for chicken embryos. The embryos were staged according to Tokita and Kuratani (2001; TK stages) and Hamburger and Hamilton (1951; HH stages) for turtle and chicken, respectively. Embryos were fixed with 4% paraformaldehyde in phosphate-buffered saline or Serra's fixative.

### In situ hybridization

Antisense and sense RNA probes were generated by in vitro transcription using the DIG RNA Labeling Kit (Roche Applied Science, Tokyo, Japan) according to the manufacturer's protocol. Whole-mount in situ hybridization was performed as described by Murakami et al. (2001). Section in situ hybridization was performed using Discovery XT (Ventana Automated Systems, Tucson, AZ, USA) according to the manufacturer's protocol. The stages of each embryo were matched by comparison of their thoracic anatomy (Nagashima et al. 2005). Riboprobes for *P. sinensis myogenin, Myf5*, *MyoD, MRF4*, *Pax3, Pax7*, *HGF, Met*, *Lbx1, APCDD1*, and *LEF1* were generated based on the nucleotide sequences AB480162, AB247184, AB188356, AB491206, AB188350, AB188351, AB480164, AB480165, AB472746, AB124565, and AB124566 deposited in GenBank, respectively. Riboprobes for chicken *Pax3* and *HGF* were generated based on the nucleotide sequences for AB080581 and X84045 deposited in GenBank, respectively.

### Whole-mount immunostaining and immunohistochemistry

For observation of the muscle plate, whole-mount and cryosection (12 μm) immunostaining were performed using MF20 (Developmental Studies Hybridoma Bank) as described (Murakami et al. 2001; Ohya et al. 2005;). Alexa Fluor 488-conjugated goat anti-mouse IgG (Molecular Probes, Eugene, OR, USA) was used as the secondary antibody.

### Transplantation of HGF antagonist-expressing cells

The HGF antagonist (PsHgf/NK4) cDNA sequence was cloned into pcDNA3.1 plasmids (Invitrogen, Carlsbad, CA, USA). COS-7 cells were grown in Dulbecco's-modified Eagle's medium supplemented with 10% fetal bovine serum. Cells were transfected with 2 mg of LacZ and/or *PsHgf/NK4* expression plasmids on culture dishes (BD Biosciences, San Jose, CA, USA) with Lipofectamine LTX Reagent (Invitrogen), according to the manufacturer's protocol. After removing the transfection solution, transfected cells were cultured for 24 h and then transferred into an agar-coated dish to allow the formation of cell aggregates. Cell aggregates approximately 100 μm in diameter were used for transplantation. Each aggregate was transplanted under the dermomyotome at the thoracic level of TK stage 12 embryos. The embryos were then incubated for 2 or 3 days after the operation and fixed in Serra's fixative.

## RESULTS

### Distinct configuration of turtle muscle plate

**Fig. 1:**
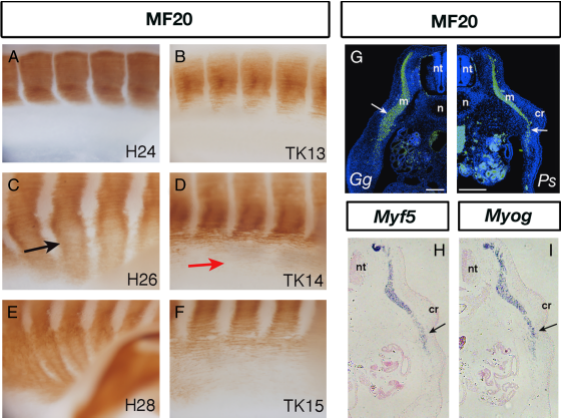
Comparison of muscle plates between *Pelodiscus sinensis* and chicken embryos. Whole-mount immunostaining of myosin heavy chain using the MF20 antibody in chicken and *P. sinensis* embryos. Hamburger–Hamilton (HH) stages 24 (A), 26 (C), and 28 (E) chicken embryos and Tokita–Kuratani (TK) stages 13 (B), 14 (D), and 15 (F) *P. sinensis* embryos were examined. The black arrow in (C) indicates muscle plate tissue extending into the lateral body wall region, which maintains its segmental organization. The red arrow in (D) shows MF20-positive myotomal fibers in the lateral body wall in a *P. sinensis* embryo. (G) Transverse sections immunostained with MF20. Note the massive and tightly packed muscle plate in the lateral body wall of an HH stage 26 chicken embryo (left), compared with the sparse myotomal cells in a TK stage 14 turtle embryo muscle plate (right). (H, I) Section in situ hybridization of TK stage 14 *P. sinensis* with a probe for *Myf5* (H) and *myogenin* (I). Arrows in (G), (H), and (I) indicate the junction of the lateral body wall and the axial part of the embryonic body. Scale bar=100 μm for (G). cr, carapacial ridge; m, myotome; n, notochord; nt, neural tube.

To understand the difference in myotome development at the thoracic level between *P. sinensis* and chicken, whole embryos were stained with MF20, a monoclonal antibody that recognizes the myosin heavy chain (Fig. 1, A–F). The staining patterns were very similar between HH stage 24 chicken and TK stage 13 *P. sinensis*, and no significant differences were found (Fig. 1, A and B). At HH stage 26 in chicken, the segmentally organized staining of myotome started to be clearly observed in the lateral body wall (Fig. 1C). On the other hand, in the *P. sinensis* at the corresponding stage (TK stage 14), MF20-positive myotomes had lost their segmental organization in the lateral body wall, and relatively fewer myotomal fibers were observed there (Fig. 1D). Similar patterns were found in older embryos (Fig. 1, E and F). Immunohistochemistry on sections (Fig. 1G) confirmed the above differences: there was a thick myotome in the chicken lateral body wall (Fig. 1G, left), whereas there was a thin and sparse myotome in *P. sinensis* (Fig. 1G, right).

### Expression patterns of genes related to muscle development are conserved in the turtle muscle plate

To identify the mechanism that produces the sparse myotomal cells in the lateral body wall of turtle embryos, expression patterns of regulatory genes functioning in muscle differentiation were compared between the two species. Unlike the previous study done with whole embryos (Ohya et al. 2006), in this study using sections, *Myf5* and *MyoD* expression were detected in the above sparse migrating cells in the lateral body wall of *P. sinensis* (Fig. 1H and data not shown). *Myogenin* and *MRF4*, which also belong to the MRFs, showed similar expression patterns between turtle and chicken embryos (Fig. 1I and data not shown), implying that the expression patterns of MRF genes alone cannot explain the unique organization of muscle plate in the lateral body wall of turtles.

**Fig. 2:**
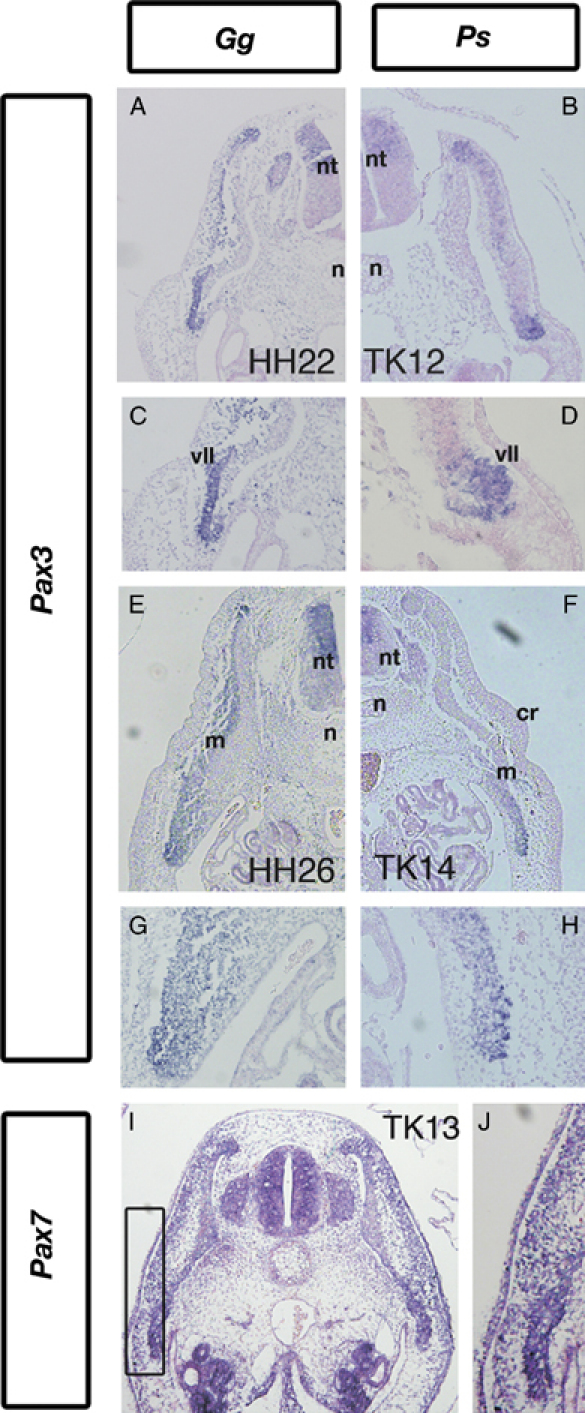
Comparison of the expression patterns of *Pax3* and *Pax7* in chicken and *Pelodiscus sinensis* embryos. Expression of *Pax3* in transverse sections through the thoracic level of Hamburger–Hamilton (HH) stages 22 (A, C) and 26 (E, G) chicken and Tokita–Kuratani (TK) stages 12 (B, D) and 14 (F, H) *P. sinensis* embryos. In each panel, the lower pictures (C, D, G, and H) are magnified views of the ventrolateral lip (VLL) region of the upper pictures (A, B, E, and F). (I) Expression of *Pax7* in transverse sections through the thoracic level of TK stage 13 *P. sinensis* embryo. (J) Higher magnification of the box in (I). cr, carapacial ridge; m, myotome; n, notochord; nt, neural tube; vll, ventrolateral lip.

Pax3 is known as a master regulatory gene for myogenic cells (Tajbakhsh et al. 1997; reviewed by Buckingham and Vincent 2009). In TK stage 12 turtle embryos, *Pax3* was expressed in the dorsomedial lips (DML) and the ventrolateral lips (VLL) of the dermomyotome, which is similar to that in chicken embryos (Fig. 2, A–D). In *P. sinensis*, however, the VLL was less epithelial than that in chicken (Fig. 2, C and D), implying accelerated delamination of the VLL in turtles. In the later stages as well, the *Pax3* expression pattern was similar between chicken and *P. sinensis* (Fig. 2, E–H), although the number of *Pax3*-expressing cells in the lateral body wall was apparently small in *P. sinensis* compared with that in chicken. Expression of *Pax7* was also detected in the DML, VLL, and dorsal dermis of TK stage 13 turtle embryos (Fig. 2, I and J). This pattern of *Pax7* expression was similar to that of the chicken embryo (Ahmed et al. 2006). These results show that it is not the expression patterns of *Pax3* and *Pax7*, but the *number* of *Pax3*-positive cells that correlates with the poor development of muscle plate and accelerated delamination of the VLL in turtles.

### Unique expression of *HGF* in turtle embryo

The sparse morphology of the muscle plate and the accelerated collapse of the epithelial state of the VLL observed in the lateral body wall of the turtle embryos were reminiscent of the development of limb muscles. In this process, the precursor cells de-epithelialize from the VLL of dermomyotome at the neck and limb level and migrate a long distance toward the limb bud, where they differentiate into mature myotubes. These cells are termed migrating muscle precursors (MMPs; reviewed by Bladt et al. 1995; Daston et al. 1996; Dietrich et al. 1999;: also see Kusakabe and Kuratani 2005, 2007). HGF and Met signaling are both required for delamination from the epithelial VLL. Ligand-encoding *HGF* is expressed in the pathway and target sites of these MMPs, and receptor Met is distributed in the VLL at all the axial levels in mouse and chicken embryos. To elucidate the involvement of such signaling in the early delamination of the VLL at the interlimb level of turtle embryos, we next observed the expression patterns of these genes.

**Fig. 3:**
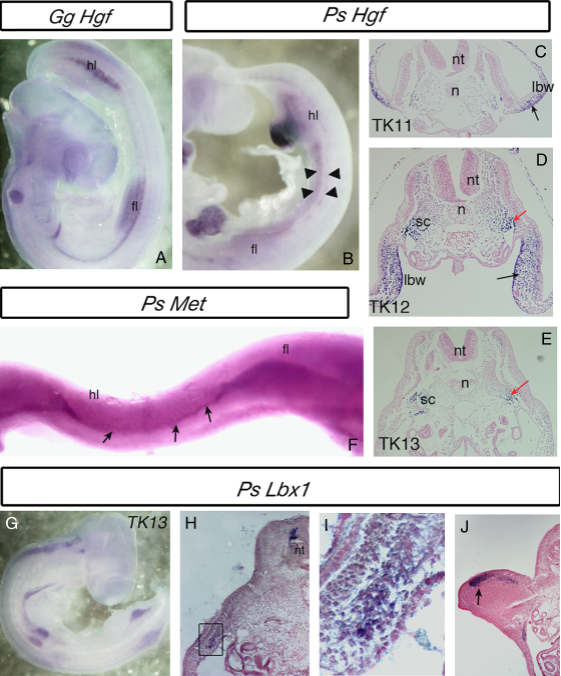
Expression of *HGF, Met*, and *Lbx1* in *Pelodiscus sinensis* embryos. Expression of *hepatocyte growth factor* (*HGF*) gene in Hamburger–Hamilton (HH) stage 17 chicken (A) and Tokita–Kuratani (TK) stage 12 *P. sinensis* (B). Arrowheads indicate the *HGF* expression in the interlimb region of turtle. Expressions of *HGF* in transverse sections at the thoracic level of TK stages 11 (C), 12 (D), and 13 (E) *P. sinensis* embryos. The black arrows indicate expression in the lateral body wall and red arrows indicate expression in the sclerotome. (F) Expression of *Met* in TK stage 10 *P. sinensis*. Arrows indicate the expression in the VLL. (G) Expression of *Lbx1* in TK stage 13 *P. sinensis*. Expression of *Lbx1* in transverse sections of TK stage 13 *P. sinensis* at the thoracic (H, I) and the forelimb level (J). (I) Higher magnification of the boxed region in (H). fl, forelimb; hl, hind limb; lbw, lateral body wall; n, notochord; nt, neural tube; sc, sclerotome.

The *P. sinensis* embryo showed a unique expression pattern of *HGF* that so far has not been reported in other amniotes. Thus, in chicken embryos *HGF* expression was restricted to the limbs and neck region, and the interlimb levels did not show any expression of *HGF* (Fig. 3A), confirming a previous study by Heymann et al. (1996). However, in *P. sinensis HGF* transcripts were detected not only in the limb buds but also at the interlimb levels, forming two longitudinal bands along the rostrocaudal axis (Fig. 3B, arrowheads). By in situ hybridization on histological sections, these turtle-specific *HGF* expressions were confirmed in sclerotome as well as in the lateral body wall (Fig. 3, C–E). The *HGF* expression in the lateral body wall started at TK stage 11 and became downregulated before TK stage 13 (Fig. 3, C–E). On the other hand, expression in the sclerotome started at TK stage 12 and continued up to stage 14 (Fig. 3, C–E and data not shown). Expression of the receptor *Met* was identified in the VLL at both limb and interlimb level in TK stage 10 *P. sinensis* embryos by whole-mount in situ hybridization (Fig. 3F), although the expression level was very low. The latter expression pattern was consistent with those of other amniotes (Heymann et al. 1996; Yang et al. 1996;), which are also very weak (Myokai et al. 1995).

*Lbx1* is expressed specifically in MMPs, and plays an important role in conferring their identity (Schäfer and Braun 1999; Brohmann et al. 2000;). In the TK stage 13 *P. sinensis* embryo, some of the cells derived from somites showed *Lbx1* expression in the lateral body wall, although the expression level was much lower than that in the occipital, cervical, and limb regions (Fig. 3, G–J).

### Turtle-specific *HGF* expression is necessary for CR formation

**Fig. 4:**
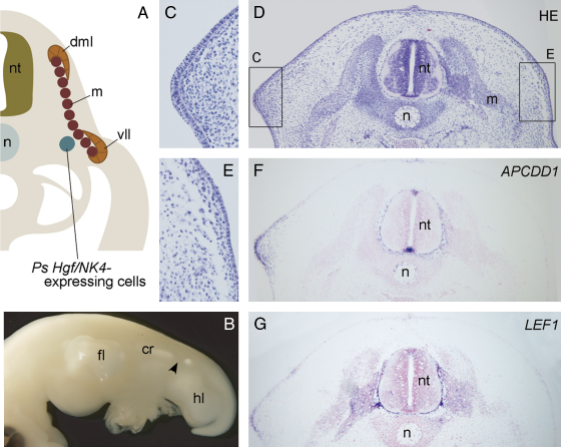
Hepatocyte growth factor (HGF) antagonist inhibited carapacial ridge (CR) formation. (A) A diagram showing the transplantation of COS-7 cells expressing *PsHgf/NK4*, an antagonist of HGF, into the *Pelodiscus sinensis* embryos. Aggregates of transfected cells were transplanted medially to the VLL of Tokita–Kuratani (TK) stage 12 embryos. (B) Three days after transplantation, a notch in the CR (arrowhead) appeared at the site of implantation, showing the local arrest of CR formation. Transverse sections of the same embryo shown in (B) at the arrowhead level. (C) Enlargement of the control side of the embryo in (D) shows histology of the intact CR. (E) Enlargement of the operated side of the same embryo. Note that the intact CR comprises a thickened epidermis covering the accumulated mesenchyme (C), whereas on the operated side these characteristic features of the CR have been lost (E). *APCDD1* (F) and *LEF1* (G) expression in the adjacent section to (D). CR-specific gene expression was not detected in the operated side. cr, carapacial ridge; dml, dorsomedial lip; fl, forelimb; hl, hind limb; m, myotome; n, notochord; nt, neural tube; vll, ventrolateral lip.

To investigate the roles of turtle-specific *HGF* expression, we implanted aggregates of COS-7 cells expressing *P. sinensis Hgf/NK4*, a specific antagonist of HGF (Date et al. 1997), under the dermomyotome of *P. sinensis* embryos at TK stage 12 (Fig. 4A). After 2–3 days of incubation, the histological configuration of the CR—the thickened ectoderm and underlying condensed mesenchyme—was lost on the operated side at the level of grafts (8/8 embryos studied; Fig. 4, B–E). By contrast, no defect was found either on the control side or in embryos implanted with a COS-7 cell aggregate expressing *LacZ* (0/3 embryos; data not shown). Expressions of CR marker genes, *APCDD1, lymphocyte enhancer factor* (*LEF1*) and *cellular retinoic acid-binding protein* (*CRABP)1* (Kuraku et al. 2005), were also abolished in the operated side, suggesting that CR formation had been arrested totally by the HGF inhibitor (Fig. 4, F and G and data not shown). In contrast, no significant change was observed in the muscle plate morphology in the lateral body wall (Fig. 4D and data not shown).

## DISCUSSION

**Fig. 5:**
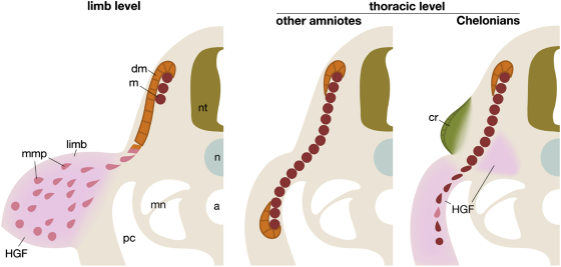
Function of hepatocyte growth factor (HGF) in turtle embryogenesis. At the limb bud level of the amniote embryo (left), *HGF* expressed in the limb mesenchyme causes delamination of the VLL and produces migrating muscle precursors (MMPs). MMPs specifically expressing *Lbx1* migrate into the limb bud and differentiate into myoblasts to form limb muscles. At the interlimb level of nonchelonian amniotes (middle), *HGF* is not expressed and somitic cells migrate into the lateral body wall, maintaining the epithelial state of the VLL and a packed sheet-like organization of myotome. These somitic cells do not express *Lbx1* and produce massive muscle plate in the lateral body wall. By contrast, at the interlimb level of the turtle embryo (right), HGF in the lateral body wall is supposed to induce early de-epithelialization of the VLL, which together with a decrease in the myogenic activity of Myf5 would result in the formation of fewer myotomal cells and a thinner muscle plate. Some of these somitic cells express *Lbx1*, but it is unclear whether these cells co-express MRFs. *HGF* expressed in the sclerotome induces the CR in the axial dermis. a, dorsal aorta; cr, carapacial ridge; dm, dermomyotome; m, myotome; mmp, migratory muscle precursors; ms, mesonephros; n, notochord; nt, neural tube; pc, pleural cavity.

Here we carried out whole-mount immunostaining using the antimyosin antibody MF20 and observed the pattern of myotomal cells migrating into the lateral body wall. In *P. sinensis* embryos, the muscle plate was comprised of fewer MF20-positive myotomal cells than in chicken embryos, and these turtle cells were distributed sparsely in the lateral body wall. On the other hand, in chicken embryos the muscle plate was made up of densely organized myotomal cells (Figs. 1 and 5, middle). To identify the developmental mechanism behind the unique thoracic myotome development in turtles, we first examined the expression patterns of the MRFs. Unlike the previous study (Ohya et al. 2006), using section-based in situ hybridization we could find the expression of *Myf5* and *MyoD* in cells migrating into the lateral body wall of *P. sinensis* in a pattern similar to that in chicken embryos, although there were much fewer cells expressing these genes in *P. sinensis* (Fig. 1H and data not shown). Other MRFs and their upstream transcriptional factors showed expression patterns in *P. sinensis* that were similar to those in other amniotes (Figs. 1I and 2 and data not shown).

The expression patterns of these genes do not explain the turtle-specific configuration of the muscle plate per se, but the abnormal feature of the *Myf5* gene in turtles might do so. Namely, the turtle *Myf5* shows a nucleotide deletion in the transactivation domain as well as a splicing variant potentially functioning as a dominant negative form (Ohya et al. 2006), suggesting that *Myf5* might be responsible, in part, for the fewer myocytes in the turtle. The earlier de-epithelialization of the VLL in turtles is supposed to further accelerate a decrease in the number of myoblasts in hypaxial domain, because the VLL is the source of ventral myoblasts (Gros et al. 2004; reviewed by Christ and Ordahl 1995; Scaal and Christ 2004; Christ and Scaal 2008;). Therefore, we analyzed the expression patterns of *HGF* and its receptor *Met*, which are known to play important roles in the delamination and migration of MMPs from the VLL at occipital, cervical, and limb levels in amniotes (Fig. 5, left; Daston et al. 1996; Dietrich et al. 1999;). Mice lacking either the ligand or its receptor are devoid of MMP derivatives, such as limb muscles, diaphragm, and intrinsic tongue muscles (Bladt et al. 1995; Maina et al. 1996; Yang et al. 1996; Dietrich et al. 1999;). Furthermore, ectopic application of exogenous HGF adjacent to chicken somites induces delamination of the VLL (Brand-Saberi et al. 1996; Heymann et al. 1996;).

In *P. sinensis* embryos *HGF* was expressed uniquely in the sclerotome and lateral body wall at the interlimb level (Fig. 3) in addition to the other expression domains such as limb buds and cervical region, which are similar to those in other amniotes. The *HGF* expression in the turtle lateral body wall implies that HGF might induce early delamination in the VLL and the resulting decrease in myoblast numbers and sparse configuration of the muscle plate (Fig. 5, right). To investigate this hypothesis, a knockdown of *HGF* function in the lateral body wall is indispensable. However, in our hands this experiment was not successful because we were unable to introduce exogenous genes to mesenchymal tissues and the turtle lateral body wall was too thin to manipulate successfully.

Do these sparse myotomal cells have an identity as MMPs? To address this question, we investigated the expression of *Lbx1*, which is usually expressed in all MMPs in vertebrates (Schäfer and Braun 1999; Brohmann et al. 2000; Neyt et al. 2000;). Some cells in the turtle lateral body wall, which are supposed to be derived from somites, showed low levels of *Lbx1* expression (Fig. 3, H and I), implying that these cells could have gained an MMP-like property via the HGF/Met-signaling pathway. Meanwhile, *Myf5* expression was detected in the muscle plate of *P. sinensis* embryos (Fig. 1H). According to previous reports (reviewed by Bladt et al. 1995; Daston et al. 1996; Dietrich et al. 1999;), MMPs do not express MRFs during migration. Further detailed analyses are required to elucidate the character of these sparse cells in the turtle lateral body wall.

As described above, rib development depends totally on myotome development. In many mouse models carrying mutations in genes related to myotome formation, the distal portions of the ribs tend to be severely affected (Braun et al. 1992; Hasty et al. 1993; Braun and Arnold 1995; Patapoutian et al. 1995; Zhang et al. 1995; Yoon et al. 1997; Tremblay et al. 1998; Dickman et al. 1999; Henderson et al. 1999; Grifone et al. 2005;). This indicates that the myotomal dependency of rib development would be higher toward their distal ends. It is thus plausible to assume that such deficient myogenesis in turtles would result in the axial arrest of the turtle ribs.

As another unique expression domain of *HGF*, the role of this factor in sclerotome is intriguing (Fig. 5, right). We could successfully implant cell aggregates expressing an antagonist of HGF, *Hgf/NK4*, under the dermomyotome. Unexpectedly, the inhibitor completely arrested the development of the CR, as confirmed by both histological and molecular analyses (Fig. 4). This result indicates that HGF in the sclerotome is indispensable for the formation and/or maintenance of the CR. As to CR development, involvement of the canonical Wnt pathway has been suggested from the gene expression patterns as well as functional analyses (Kuraku et al. 2005; Nagashima et al. 2007; reviewed by Kuratani et al. 2011). Although we carried out expressional analysis of *Wnt* genes, none of them has been identified as being expressed in the CR or its adjacent domain (data not shown). However, in this study we first identified HGF as a candidate for the upstream factors of this signaling cascade, because *APCDD1* expression was abolished after application of the HGF inhibitor (Fig. 4F). This expression is known to be regulated by the β-catenin/LEF1 complex, downstream of the canonical Wnt-signaling pathway (Takahashi et al. 2002; Shimomura et al. 2010;).

Actually, interactions between HGF/Met-signaling and the Wnt/β-catenin pathway have been proposed recently. In cancer cells, activation of Met by HGF induces nuclear translocation of β-catenin, leading to activation of TCF/LEF-mediated gene transcription (Danilkovitch-Miagkova et al. 2001; Monga et al. 2002; Rasola et al. 2007; reviewed by Nelson and Nusse 2004). Abrogation of *LEF1* expression after application of the HGF antagonist suggests that this gene's expression is autoregulated (Hovanes et al. 2000; Arce et al. 2006; Yoo et al. 2009;). As to the abolishment of *CRABP1* expression after this treatment (data not shown), expression of retinoic acid-binding proteins including *CRABP1* is reported to be modulated indirectly by the Wnt/β-catenin pathway (Collins and Watt 2008). Such secondary effects appear to play a role in induction of the gene in the CR as well. From these results, it is probable that acquisition of the HGF expression domain in the ancestors of turtles triggered the “invention” of the CR. At least, such a change in HGF expression must have been a prerequisite for the evolution of the CR. To confirm these possibilities, we are now trying to verify whether the HGF is sufficient to induce the CR in chicken embryos.

In our previous studies, based on the “folding theory” of turtle evolution, we have suggested that gradual, stepwise changes could have yielded the peculiar anatomical pattern of the turtle. Namely, the ribs of an ancestral animal would have first arrested developmentally in the axial part of the embryonic body, failing to penetrate into the body wall. The CR enhanced the marginal growth of the carapacial plate and fanning-out of the ribs, to encapsulate the scapular anlage dorsally. However, this scenario did not explain the hypomorphic development of the turtle trunk muscles and the de novo appearance of the CR. The data obtained in the present study tend to endorse the folding theory: poor development of myotome caused by functional change in *Myf5* and HGF expression in the lateral body wall would have resulted in the axially arrested ribs of turtles. In addition, the acquisition of an *HGF* expression domain in sclerotome would have been one background for the co-option of nuclear β-catenin signaling to form the CR, leading to the marginal growth of the carapacial primordium and the fanned-out arrangement of ribs, leading to encapsulation of the scapula (Nagashima et al. 2009). These developmental changes could be coupled but were probably not as simple in the evolution of turtles as expected previously (reviewed by Kuratani et al. 2011). Our present findings fit well not only into the morphological, but also the paleontological data (Li et al. 2008; Nagashima et al. 2009; reviewed by Kuratani et al. 2011), in which multiple steps should be assumed during turtle evolution.
